# Risk factors for cardiovascular events among Asian patients without pre-existing cardiovascular disease on the renal transplant wait list

**DOI:** 10.7603/s40602-015-0001-0

**Published:** 2015-04-15

**Authors:** Wong Ningyan, Chin Chee Tang, Tee Ping Sing, Khin Lay Wai, Angela S. Koh, Kee Yi Shern, Terence, Tan Wei Chieh, Jack

**Affiliations:** 1 Department of Internal Medicine, Singapore General Hospital, Singapore, Singapore; 2 Department of Cardiology, National Heart Centre Singapore, Singapore, Singapore; 3 Department of Renal Medicine, Singapore General Hospital, Singapore, Singapore; 4 Duke-National University Singapore Graduate Medical School, Singapore, Singapore; 5 Investigational Medical Unit, Dean’s Office, Singapore, Singapore; 6 Department of Surgery, Yong Loo Lin School of Medicine, National University of Singapore, Singapore, Singapore

## Abstract

**Introduction:**

For suitable end-stage renal failure (ESRF) patients, renal transplantation gives better long term survival and quality of life as compared to dialysis. Prior to entry into the renal transplant wait list, potential candidates are screened for the presence of cardiovascular disease. However, the waiting time on the transplant list is long, and interval screening for cardiac fitness for surgery is not well defined. We aim to study the risk factors for the development of a cardiovascular event (CVE) and the time interval from recruitment to onset of a CVE that resulted in their removal from the transplant wait list.

**Methods:**

A retrospective study of all patients registered under the cadaveric renal transplant waiting list in Singapore General Hospital (SGH) from 16^th^ April 1987 to 31^st^ October 2010. We identified patients who developed a CVE among this cohort. We compared the demographics and clinical characteristics of patients who experienced a CVE versus those who did not. Univariable and multivariable cox regression were performed to investigate the significant variables for the development of a CVE. The time to development of CVE was estimated using Kaplan Meier estimation and log-rank test was used to compare the time to CVE between those with diabetes mellitus and those without.

**Results:**

1265 patients were enrolled in this study. 273 patients dropped out of the wait list due to medical reasons or death, of which 38.8% were due to CVE. The mean and median time duration from recruitment into the waiting list to development of a CVE was 14.42 (95% CI 13.72 to 15.11) and 15.69 (95% CI 13.86 to 17.51) years respectively. For patients with diabetes mellitus, this was 8.22 (95% CI 6.30 to 10.14) and 8.16 (95% CI 4.95 to 11.36) years respectively. Factors associated with an increased risk of developing a CVE included male gender (adjusted HR 2.21, 95% CI 1.43 to 3.41, p<0.001), presence of diabetes mellitus (adjusted HR 5.13, 95% CI 2.85 to 9.24, p<0.001) and patients who were either not working or working part-time as compared to their full-time counterparts (adjusted HR 1.76, 95% CI 1.14 to 2.72, p=0.010). In addition, hazard ratio for CVE significantly increased with advancing age quartile (p<0.001 by log rank test for trend).

**Conclusion:**

A significant proportion of patients exited from the renal transplant wait list due to a CVE. Being male, age 37 years old or more, presence of diabetes mellitus and non-working or part-time workers as compared to full-time workers were found to increase the risk of developing a CVE during the wait period for transplantation. The presence of diabetes mellitus significantly shortened the time to development of a CVE.

## Introduction

For suitable end-stage renal failure (ESRF) patients, renal transplantation gives better long term survival and quality of life as compared to dialysis.(1-6) However the wait for a suitable renal transplant is usually long, with a median waiting time of 9.44 years for a cadaveric renal transplant in Singapore.(7)During this period, this group of patients can develop significant cardiovascular diseases.

In Singapore General Hospital (SGH), patients have to undergo a thorough evaluation and a battery of screening tests prior to entry into the cadaveric renal transplant list. Patients will be excluded if they have any of the following: cardiovascular disease with ongoing symptoms, coronary artery disease with or without revascularization, untreated severe valvular heart disease, untreated arrhythmia; cerebrovascular disease; peripheral vascular disease with ongoing ischaemic symptoms, critical arterial stenosis, gangrene, non-healing ulcer or aneurysm; history of malignancy; human immunodeficiency virus infection; active hepatitis C with detectable viraemia, chronic active hepatitis or liver cirrhosis; any active infection that has not resolved or tuberculosis within the last 6 months; severe chronic lung disease; active inflammatory gastrointestinal disease; active systemic lupus erythematosus; active psychiatric issues; history of non-compliance within the last 6 months. Cardiovascular evaluation includes a chest x-ray (CXR) and electrocardiogram (ECG) for all patients. For those above 60 years of age or has diabetes mellitus, a myocardial perfusion scan will be included. Patients with diabetes mellitus who have a left ventricular ejection fraction (LVEF) of more than 40 percent are required to undergo a coronary angiogram (those with LVEF of less than 40 percent are excluded from entry into the transplant list). A cardiologist evaluation is required for all patients with diabetes mellitus or those with abnormal screening tests.

Although a strict selection criteria to determine medical fitness before recruitment into the cadaveric renal transplant list exists, over time the medical fitness of a patient inevitably deteriorates. This may then result in their removal from the transplant wait list. Of the medical conditions that patients with chronic kidney disease encounter, cardiovascular events have the highest incidence and prevalence.(8-10)

Currently, there is a paucity of information with regards to the cardiovascular event (CVE) rates among patients on the renal transplant list. In addition, there is also a lack of consensus for effective interval cardiovascular screening among this group of patients.

Hence our study had the following objectives. We evaluated the proportion of renal patients in a single centre (Singapore General Hospital) that developed a CVE which subsequently resulted in their removal from the cadaveric renal transplant list. Secondly, among the patients on the renal transplant waiting list, we determined the factors that were associated with developing a CVE. Lastly, we evaluated the time to development of CVE, comparing between those with diabetes mellitus and those without.

## Methods

We conducted this retrospective study by analyzing data from the SGH renal transplant waiting list. The database contained demographical information and clinical characteristics of the wait-listed patients. Data forms were completed by the patient’s primary physician (nephrologist) and a trained data extractor. Random case record sampling was performed by the study team to assess the accuracy of the data.

We conducted a retrospective review of all patients registered under the renal transplant waiting list in SGH during the period 16^th^ April 1987 to 31^st^ October 2010. We identified patients who became inactive following a CVE. Inactive patients were defined as those that were excluded from the list due to medical events, death, advanced age, received a kidney transplant and others. Other reasons included patients that opted out of the registry due to personal reasons and those that experienced a medical event but were still pending further medical evaluation during the time of data collection.

CVE was defined as ischaemic heart disease (IHD), nonischaemic cardiac events and cerebrovascular accidents (CVA). IHD included patients who experienced an acute coronary syndrome, had coronary artery stenosis of greater than 50 percent in at least one epicardial coronary artery on angiography and history of revascularization. Non-ischaemic cardiac events included non-ischaemic cardiomyopathies of LVEF less than 50 percent, severe valvular disease and untreated arrhythmias, while CVA included both ischaemic and haemorrhagic cerebrovascular accidents. We compared the demographical and clinical characteristics of patients who experienced a CVE versus those who did not, and identified the risk factors for developing a CVE. Demographical characteristics included age, gender, race, marital and employment status. Clinical characteristics included type of dialysis, type of underlying kidney condition, ECG evidence of left ventricular hypertrophy (LVH), CXR evidence of cardiomegaly and presence of diabetes mellitus.

Categorical variables were presented as numbers and percentages. Demographical and clinical characteristics were compared using chi-square test, Fisher’s exact test, or the unpaired t-test, as appropriate. A two-sided p value of less than 0.05 was considered to indicate statistical significance. Univariable and multivariable cox regression were performed to investigate the independent significant variables for the development of cardiovascular events. The time to development of CVE was estimated using Kaplan Meier estimation. Log-rank test was used to compare the time to CVE between those with diabetes mellitus and those without. Statistical analyses were performed using SPSS version 19.0 (IBM Corporation).

## Results

**Figure 1.**
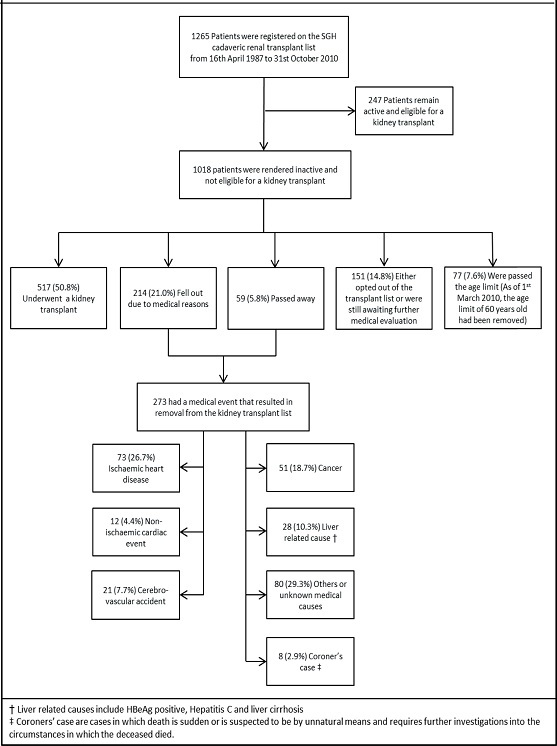
Reasons for falling out of the cadaveric renal transplant list.

A total of 1265 patients were enrolled in our study (*Figure* 1). At the time of data collection, 1018 patients had become inactive while 247 remained active and eligible for a transplant on the renal transplant list. Reasons for falling out included having undergone a successful kidney transplant, experienced a medical event that rendered them ineligible to remain on the transplant list, passed away, personal reasons (e.g. personal decision to be removed from the transplant list), or those who experienced a medical event and were still pending further medical evaluation to determine their suitability to remain on the transplant list during the time of data collection. The age limit was set at 60 years old in the past but this exclusion had since been removed from 1st March 2010 onwards. 273 patients fell out due to medical reasons or death, of which 38.8% fell out due to a CVE (26.7% from IHD, 4.4% from non- IHD cardiac causes and 7.7% from CVA). Other significant medical reasons for falling out included cancer (18.7%) or a liver related cause (10.3%).

*Table* 1 compares the demographical and clinical characteristics of patients with and without CVE. Of note, only 81 out of the 1265 patients enrolled in this study had diabetes mellitus. This could be due to the fact that many patients with diabetes mellitus had already experienced cardiovascular events or had other chronic medical conditions that rendered them ineligible for entry into the wait list right at the onset.

As depicted in *Table* 2, using multivariable cox regression analysis, significant independent variables for CVE included male gender (adjusted HR 2.21, 95% CI 1.43 to 3.41, p<0.001), presence of diabetes mellitus (adjusted HR 5.13, 95% CI 2.85 to 9.24, p<0.001) and working part-time or not-working as compared to working full-time (adjusted HR 1.76, 95% CI 1.14 to 2.72, p=0.010). In addition, hazard ratio for CVE significantly increased with advancing age quartile (p<0.001 by log rank test for trend) after adjusting for other significant variables such as gender, presence of diabetes mellitus and employment status in the final fitted cox regression model. The adjusted hazard ratios for the 3 highest age quartiles were significantly higher than the reference lowest age quartile. The adjusted hazard ratio estimates for the age quartile range of 37 to 42 years old, 43 to 48 years old and more than 48 years old were 3.74 (95% CI 1.85 to 7.55, p<0.001), 4.95 (95% CI 2.44 to 10.01, p<0.001) and 8.19 (95% CI 3.85 to 17.42, p<0.001) respectively.

**Figure 2.**
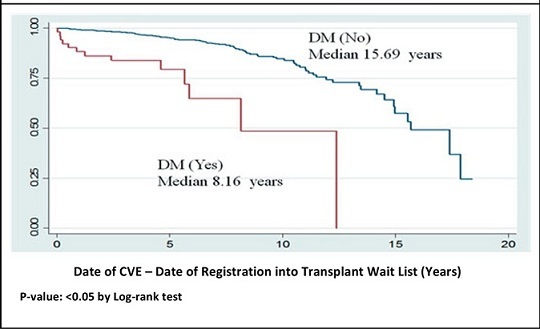
Kaplan-Meier estimate of time to CVE among those with and without DM.

We studied the time duration from registration into the wait list to development of a CVE. Using Kaplan-Meier estimation, the mean and median time to event were 14.42 (95% CI 13.72 to 15.11) and 15.69 (95% CI 13.86 to 17.51) years respectively. As shown in *Figure* 2, log-rank test was used to compare the time to CVE between those with diabetes mellitus and those without. The mean and median time to event were 8.22 (95% CI 6.30 to 10.14) and 8.16 (95% CI 4.95 to 11.36) years respectively for patients with diabetes mellitus.

## Discussion

Our study demonstrated that, among those that exited the cadaveric renal transplant list due to medical reasons, 38.8% were due to a CVE. Secondly, factors associated with an increased risk of developing a CVE included male gender, age 37 years old or more, presence of diabetes mellitus and working part-time or not-working as compared to working full-time. Lastly, the presence of diabetes mellitus significantly shortened the time to development of a CVE.

The most common medical reason for exiting the cadaveric renal transplant list is cardiovascular events (38.8%). This high prevalence of CVE can be explained by the increased risk of arterial vascular disease (atherosclerosis and arteriosclerosis), cardiomyopathy, as well as accelerated cardiac valve calcification in patients with chronic kidney disease on dialysis. Atherosclerosis in this group of patients typically manifests itself with the cardiovascular complications of ischaemic heart disease, cerebrovascular disease and peripheral vascular disease. (11-13) Arteriosclerosis causes a reduction in arterial compliance and it itself is an independent risk factor for CVE in dialysis patients.(14,15) Cardiomyopathy, in the form of systolic and diastolic dysfunction, results from the pressure overload from hypertension and arteriosclerosis, as well as volume overload from chronic anaemia, fluid overload, uraemia and occasionally hyper-functioning arteriovenous fistulas.(16) Cardiac valvular calcification results from the abnormal calcium and phosphate metabolism and increased mechanical stress on valve cusps in patients on dialysis.(17-20) Clinically this group of patients are prone to accelerated coronary and peripheral arterial disease, left ventricular hypertrophy and cerebrovascular accidents. Development of early degenerative valvular heart disease like aortic stenosis is also common.

We compared the rate of CVE among renal transplant wait list patients with that from other studies. In a study done by John S. Gill et al in British Columbia, 602 patients on the renal transplant wait list were included between July 1998 and October 2001.(21) Among these patients, 69 experienced a CVE (excluding patients who developed a CVE after transplantation) and 33 were removed from the wait-list for non-cardiac reasons. Hence, 67.6% (69 out of the 102 patients that either experienced a CVE or were removed from the waitlist due to non-cardiac reasons) experienced a CVE in this study as compared to ours of 38.8%. In another study done by Julian Konig et al in Germany, 267 patients were included into the transplant wait list between January 2003 and December 2006.(22) Out of the 267 patients, 41 (15.4%) experienced a major adverse cardiac event. In our study, out of the 1265 patients registered from 16th April 1987 to 31st October 2010, 106 (8.4%) experienced a CVE. One possible reason for the lower CVE rate in our centre was the inclusion of patients with pre-existing cardiovascular diseases into the renal transplant wait list for the studies done by John et al and Julian Konig et al. In our centre, patients with pre-existing cardiovascular diseases were mostly excluded from entry into the wait list.

In our study, factors associated with an increased risk of developing a CVE included male gender, increasing age quartile and the presence of diabetes mellitus. This was in keeping with the cardiovascular risk factors defined by the Framingham Heart Study. Patients who were not working or working part-time were found to be at a higher risk as compared to their full-time counterparts. Full time employment seems to predict a favorable longer term prognosis, likely as this group of patients is better educated and with a stronger socioeconomic support structure. As such they may participate better in selfcare and hence control of cardiovascular risk factors. Our findings were supported by a study done by S. Yusuf et al which investigated the rate of cardiovascular events among 150,000 adults in 17 high-, middle-, and low-income countries in the Prospective Urban Rural Epidemiologic (PURE) cohort study.(23) It showed that the incidence of major cardiovascular disease was highest in low-income countries, despite the fact that these countries had the lowest risk-factor burden. In a review of socioeconomic factors and cardiovascular disease by GA Kaplan et al (24), it had been shown consistently in both prospective and retrospective cohort studies that there was an inverse relation between cardiovascular disease and socioeconomic status (SES). However, various measures of SES had been used, including education, occupation and income or combination of these. Hence, it would be prudent to incorporate multiple indicators of SES and not rely solely on employment status if SES is to be used as a variable for development of CVE.

Although only a minority of our study patients had diabetes mellitus (81 out of 1265 patients), the presence of it significantly shortens the time to development of cardiovascular events. Median time from registration into the transplant list to developing a CVE among those without diabetes mellitus was 15.69 years. For patients with diabetes mellitus, the median time was reduced to 8.16 years. Diabetes mellitus is a major risk factor for atherosclerotic disease and considered a coronary heart disease equivalent.(11,25,26) Patients with diabetes mellitus and ESRF may have accelerated atherosclerosis as demonstrated in our study although the initial screening tests for this group of patients did not reveal overt CAD at the point of enrollment.

There are several important limitations to this study. Firstly, this was an observational, non-randomized, retrospective analysis. Therefore there might be unknown biases that could influence the results. Secondly, variables examined in this study were limited to those collected from the SGH renal transplant waiting list database, which included a total of 10 variables spanning demographical and clinical characteristics. Thirdly, there was no independent validation of outcomes; the diagnosis of a medical event (e.g. myocardial infarction) was made by the clinician on the ground, rather than an independent and consistent panel of doctors involved in the study. Lastly, a good proportion of patients who fell out of the transplant list due to medical reasons were left out as unknown medical causes and coroner’s cases (n=88, 32.2%). This group of patients could potentially alter the true proportion of patients who develop a CVE.

In conclusion, our study shows that despite a well-established cardiovascular evaluation prior to entry into the cadaveric renal transplant list, CVE continues to be the major medical reason for exiting the wait list. In these patients, studies to define the timing and modality of cardiovascular investigations will be needed.

**Table 1.** Comparison of demographic and clinical characteristics between patients with and without CVE.

	Characteristics	No (%)		P value
		CVE (Yes)	CVE (No)	
**Age (Quartiles)**	1^st^ Quartile (<37 years) 2^nd^ Quartile (37-42 years) 3^rd^ Quartile (43-48 years) 4^th^ Quartile (> 49 years)	14(13.2%) 28(26.4%) 32(30.2%) 32(30.2%)	320 (27.6%) 268(23.1%) 271(23.4%) 300 (25.9%)	0.014
**Gender**	Male Female	65(61.3%) 41(38.7%)	579 (50.0%) 580 (50.0%)	0.025 -
**Race**	Chinese Malay Indian Others	87(82.1%) 15(14.2%) 3(2.8%) 1(0.9%)	917(79.1%) 179(15.5%) 51(4.4%) 12(1.0%)	0.854
**Marital status**	Married Single/ Widowed/ Separated or Divorced	85(80.2%) 21(19.8%)	838(72.3%) 321(27.7%)	0.080 -
**Employment**	Full-time Part-time/ Unemployed/Home/ Not-stated	58(54.7%) 48(45.3%)	721(62.2%) 438(37.8%)	0.129 -
**Type of Dialysis**	Haemodialysis Peritoneal Dialysis	101(95.3%) 5(4.7%)	1072 (92.5%) 87(7.5%)	0.290 -
**Type of Kidney Condition**	Chronic Glomerulonephritis Not Chronic Glomerulonephritis	59(55.7%) 47(44.3%)	719(62.0%) 440(38.0%)	0.197 -
**ECG evidence of Left**	LVH	25(23.6%)	153(13.2%)	0.003
**ventricular hypertrophy**	No LVH	81(76.4%)	1006 (86.8%)	-
**Chest X-ray evidence of**	Cardiomegaly	31(29.2%)	228(19.7%)	0.019
**Cardiomegaly**	No Cardiomegaly	75(70.8%)	931(80.3%)	-
**Diabetes mellitus**	Yes No	16(15.1%) 90(84.9%)	65(5.6%) 1094(94.4%)	<0.001 -

**Table 2.** Multivariable cox regression analysis for variables associated with CVE.

	Characteristics	Adjusted Hazard Ratio	95% CI	P value
Age	l^st^ quartile (<37 years) 2^nd^ quartile (37-42 years) 3^rd^ quartile (43-48 years) 4^th^ quartile (≥49yrs)	1.00 3.74 4.95 8.19	- (1.85,7.55) (2.44,10.01) (3.85,17.42)	- <0.001 <0.001 <0.001
Gender	Male Female	2.21 1.00	(1.43,3.41) -	<0.001 -
Diabetes mellitus	Yes No	5.13 1.00	(2.85,9.24) -	<0.001 -
Employment status	Part-time/House duties/Not working/Not-stated Full-time workers	1.76 1.00	(1.14,2.72) -	0.010 -
